# Primary care physicians’ satisfaction after health care reform: a cross-sectional study from two cities in Central Java, Indonesia

**DOI:** 10.1186/s12913-019-4121-2

**Published:** 2019-05-08

**Authors:** Chatila Maharani, Desie Frihandini Afief, Dorothea Weber, Michael Marx, Svetla Loukanova

**Affiliations:** 1 Heidelberg Institute of Global Health, Medical Faculty and University Hospital, University of Heidelberg, Heidelberg, Germany; 2 Department of Public Health, Universitas Negeri Semarang, Semarang, Indonesia; 3 Public Health Unit, Central Java Provincial Health Office, Semarang, Indonesia; 4 Institute of Medical Biometry and Informatics, University of Heidelberg, Heidelberg, Germany; 5 Department of General Practice and Implementation Research, University of Heidelberg, Heidelberg, Germany; 6 Department of General Practice and Health Services Research, Marsilius-Arkaden, Im Neuenheimer Feld 130.3, Turm West, 3 OG, Raum 03.303, D-69120 Heidelberg, Germany

**Keywords:** Job satisfaction, general practitioners, primary health care, health care reform, national health insurance

## Abstract

**Background:**

In 2014, Indonesia launched a mandatory national health insurance system called Jaminan Kesehatan Nasional (JKN). The reform introduced new conditions for primary care physicians (PCPs) that could influence their job satisfaction. This study assessed PCPs’ satisfaction and its predictors in two cities in Central Java, Indonesia, following the reform.

**Methods:**

In this exploratory, cross-sectional study, we recruited 276 PCPs from the selected area. The data were all collected in 2016 using self-report questionnaires and interviews. PCPs’ satisfaction was measured using a modified version of the Warr-Cook-Wall Job Satisfaction Scale which contains 19 items and uses a Likert-type response scale. Analysis of variance, the Kruskal-Wallis H test, both with Bonferroni corrections for post hoc testing, and Cochran–Mantel–Haenszel tests were used to compare overall job satisfaction between participant groups. We used simple and multiple linear regression analyses to identify the predictors of PCP satisfaction. Furthermore, a logistic regression analysis for binary outcome was applied to model the PCPs intention to leave practice.

**Results:**

PCPs’ mean overall satisfaction level was 3.19 out of 5. They tended to be very satisfied with their relationship with colleagues, working hours, and physical working conditions. However, the PCPs were dissatisfied with the new referral system, the JKN health services standards, and JKN policy. The factors significantly associated with job satisfaction (*p* <  0.001) included type of practice, performance of managerial tasks, and PCPs’ perceptions of and experiences with patients. PCP satisfaction was negatively associated (*p* = 0.004) with PCPs’ intention to leave their practice.

**Conclusions:**

The PCPs investigated in these two cities in Central Java had moderate satisfaction after the Indonesian health care reform. PCPs who worked in solo practices, performed managerial tasks, and had good experiences with patients tended to have higher satisfaction scores, which in turn prevented them from developing an intention to leave their practice. The three aspects that PCPs with which most dissatisfied were related with the JKN reform. Because of that, the government and BPJS for Health should aim to improve the JKN system in order to increase PCPs’ satisfaction.

**Electronic supplementary material:**

The online version of this article (10.1186/s12913-019-4121-2) contains supplementary material, which is available to authorized users.

## Background

Recently, several Asian countries, such as Taiwan, China, and Thailand [1–3], have implemented health system reforms for achieving universal coverage. Similarly, on 1 January 2014, Indonesia implemented mandatory national health insurance for all citizens, called Jaminan Kesehatan Nasional (JKN). This was an effort to improve insurance coverage in the country—in 2012, only 62.1% of the Indonesian population had health insurance under a variety of different schemes; the remainder of the population was not covered by any form of health insurance [4].

The new system introduced a variety of new conditions for primary care physicians (PCPs). Before the health system reform, majority of the primary health care (PHC) payment system relied on a retrospective fee-for-service (FFS) system, and most services were paid out-of-pocket. The payment system also did not force patients and PCPs to follow the regulations of the tiered referral system [4]. Only a small portion of PCPs (5.1%) practiced as family physicians for PT. Askes (health insurer for civil servants) in 2012 and were paid by capitation [5]. Furthermore, under the system, the majority of private PHC facilities did not provide preventive or promotive health services.

The JKN reformed the payment system into a retrospective capitation system. Nowadays, PHC facilities must manage their income based on capitation not only for curative and rehabilitative services but also for preventive and promotive services. The reform also introduced, among other procedures, a more strictly tiered referral system and defined the standard non-specialist diseases that had to be treated in PHC facilities. Several new health programmes were launched, such as home visits, medical history screening, disease management programs, and counter-referral programs. PT. Askes was renamed *Badan Penyelenggara Jaminan Sosial* (BPJS) for Health, and became the sole payer under the JKN. The BPJS for Health evaluates PHC facilities’ performance and, at the beginning of 2016, implemented a pay-for-performance (P4P) system for public PHC facilities in provincial capitals [4, 6–12].

Although the Indonesian government has stated that the PCP ratio per 1000 population was sufficient to serve the entire population of Indonesia [4], the ratio falls below that recommended by the World Health Organization, 1 physician per 1000 population [13]. Specifically, the PCP ratio per 1000 population was 0.16 in 2015 [14]. Thus, although the number of physicians in Indonesia has been increasing, it still falls well short of the population growth [13]. This condition need to be considered, because job satisfaction predicted the physician intention to leave practice [15].

The change in the work conditions that follows health system reform can influence physicians’ satisfaction [16, 17]. A Chinese study investigating satisfaction following implementation of universal health coverage confirmed this [18]. However, there has been previous research on PCPs’ satisfaction in Indonesia before the reform. Most of these studies have focused only on the measurement of physician incomes under the capitation payment system created by PT. Askes before the JKN implementation. These studies tended to show that PCPs were rather dissatisfied with this capitation system [19–21]. Because PCPs’ satisfaction is associated with healthcare quality [22], there is a need to analyse it within the reform framework.

To investigate PCP satisfaction in Indonesia, we chose the Semarang Municipality and Demak Regency of Central Java Province as study areas. In Semarang municipality, the capital city of Central Java, has the highest number of physicians (i.e., general pratitioners) and public health officers in this province [23]. However, the ratio of physicians to the population in the province was not high, at only 0.14 per 1000 population [14, 24]. The health status of the populations of these regions were regarded as poor based on select indicators. Specifically, in 2014, the incidence rates (IRs) of Dengue haemorrhagic fever (DHF) per 100,000 population were 98.57 (Semarang Municipality) and 36.26 (Demak Regency), both of which were higher than the provincial average (32.95) [25]. Semarang Municipality was also included in 2013 as one of the five cities with the highest maternal mortality rate (29 cases) [26]. It also had the fourth highest mortality rate for children under the age of 5 (305 cases in 2014) and the highest number of new human immunodeficiency virus (HIV) cases (108 of 1399 cases) [25] in the province.

In light of the health system reform, it is necessary to consider the needs of health workers—especially PCPs, who work to serve patients as well as boost the overall health conditions of the surrounding areas. It is especially necessary to examine their job satisfaction and intention to leave. Therefore, this study assessed PCPs’ overall level of job satisfaction and its various aspects, as well as identified the predictors of satisfaction and PCPs’ intention to leave their practice in two cities in Central Java, Indonesia, after the healthcare reform of 2014. Obtaining this information can help us in forming recommendations for the government on how to improve conditions for PCPs following the reform.

## Methods

### Design and Setting

This exploratory, cross-sectional study was conducted in PHC facilities in two cities in Central Java, Indonesia–Semarang Municipality and Demak Regency. Both these cities are coordinated by the Semarang Main Branch Office of BPJS for Health.

### Participants

The participants were PCPs who worked in PHC facilities, namely, public health centres with or without inpatient care, private PHC clinics (usually served by 2 or more physicians), and solo practices. The study area contained 381 PCPs in total. We used purposive sampling, a nonprobability sampling technique wherein we select participants based on their specific characteristics [27]. The characteristics were the length of work in the PHC facilities and the length of the contract between BPJS for Health and the PHC facilities. The participants were PHC physicians who had worked for more than three months. We recruited participants from PHC facilities that had a contract with BPJS for Health for at least three months. Physicians who had worked for less than three months or who had worked only as physician substitutes were excluded from the study.

The research team collected the data by visiting all PHC facilities. The addresses of these facilities were obtained from the Semarang Main Branch Office of BPJS for Health. The research team comprised students pursuing a bachelor’s or master’s degree in public health, as well as graduate students. The main researcher trained the research team before conducting the survey. We used two methods for collecting the data: self-report questionnaires and interviews. The majority of PCPs completed the questionnaire by themselves, while also being given an opportunity to ask questions of the research team. Several PCPs, however, asked to be interviewed. In these cases, a member of the research team filled in PCPs’ answers. The use of multiple data collection methods can increase the response rate and reduce the amount of missing responses to questions [28]. The data collection was conducted from April to June 2016. In all cases, non-participation was the result of refusal, being on leave (maternity or sick leave), and further specialization. Three hundred eight questionnaires were submitted, but only 276 questionnaires had complete data for satisfaction. The incomplete questionnaires were excluded.

### Instruments

The questionnaire was initially developed in English and translated into Indonesian. The questionnaire comprised 4 sections: determining the main place of practice, respondents’ characteristics, PCP satisfaction, PCPs’ intention to leave their practice. The first, second, and third sections consisted of closed-ended questions, while the fourth contained a mixture of closed- and opened-ended questions. We trialled the questionnaire in Semarang Regency with 42 PCPs, who were not included in the final sample. Following the trial, the questionnaire was discussed and revised by experts, including a health officer from the Central Java Provincial Health Office and a language expert.

#### Determining the main place of practice

In Indonesia, physicians can practise in up to three different places [29]. Only PCPs working in more than one place were asked to complete this section. The section consisted of three questions evaluating their length of work, working hours, and presence of managerial tasks in each place of practice (Additional file 1). Having a longer length of work and working hours and having managerial task to perform, resulted in a higher score. The place with the highest scores on these questions was defined as their main place of practice. If they had the same scores for two places, we chose one of them randomly for inclusion in the analysis.

#### Respondents’ characteristics

This section contained three sub-sections pertaining to PCPs’ personal characteristics, job and practice characteristics, and the PCPs’ perception and experiences with their patients (Additional file 2). The first sub-section contained questions on age, gender, and duration of work in the main place of practice. As for job and practice characteristics, we focused on practice type, average monthly income for the last three months, average number of JKN patients examined per day, average number of private insured and FFS (non-JKN) insured patients examined per day, and management responsibilities. The average monthly total income was in the currency of Indonesia, the rupiah (1 US$ = Rp. 14,285.72 in January 2019). Finally, for the PCPs’ perception and experiences with their patients, we used questions adapted from other studies on patients’ unrealistic expectations [30] and perceptions of patient aggressiveness [18]. The Cronbach’s alpha for these questions was 0.626. Three invalid questions were revised by rewording the questions.

#### PCP satisfaction

We modified a validated questionnaire called the Job Satisfaction Scale by Warr, Cook, and Wall [31]. Our modifications focused on adapting the scale to Indonesian conditions, which differ from those of the developed countries in which this questionnaire has been used previously [16, 32, 33]. In this study, we used a scoping analysis of published literature to determine suitable aspects of job satisfaction in the context of the Indonesia health system reform (not reported in this article). The questionnaire consisted of 19 aspects (or items) and utilized a Likert-type rating scale (1 = very dissatisfied to 5 = very satisfied). The Cronbach’s alpha for reliability was 0.902, and the respective questions were modified. Please see the Additional file 3.

#### Intention to leave practice

This section comprised one item of closed-ended question (a binary outcome) on PCPs’ intention to leave their main practices after JKN implementation, as well as opened-ended questions on the reasons underlying their intention to leave (Additional file 4). This sub-section was adopted from a study in England [15].

### Data analysis

**Fig. 1:**
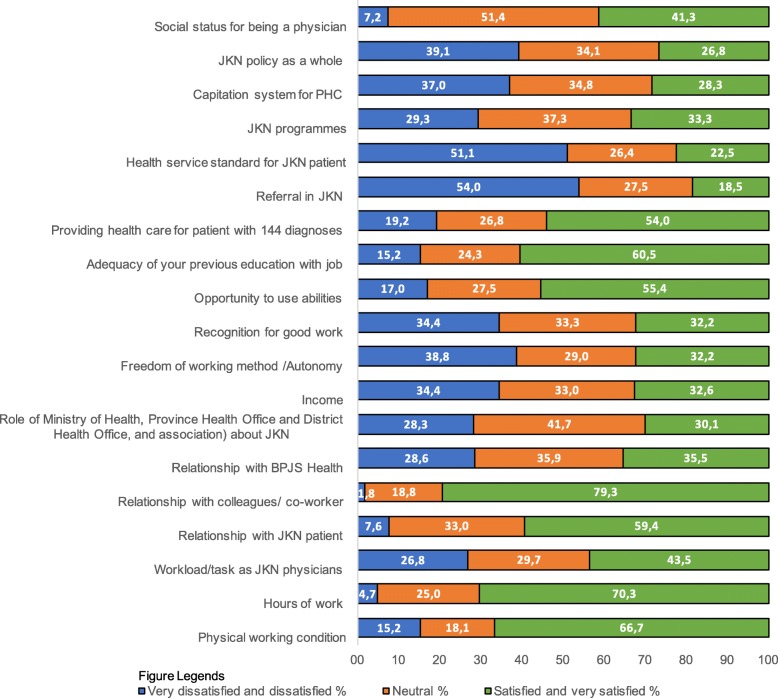
PCP satisfaction levels according to job satisfaction aspect

We obtained descriptive statistics for categorical variables (absolute and relative frequencies) and continuous variables (mean and standard deviation). To analyse overall PCP satisfaction (Tables 1, 3 and 4), we used 5-point Likert scale and treated these as continuous variable [34]. To measure overall job satisfaction, we obtained the mean score of the 19 aspects. When analysing PCP satisfaction levels according to specific aspects (Fig. 1 and Table 2)*,* we used the Likert-type data treated as (ordinal) categorical variables [34]. In these cases, we converted the 5-point Likert-type data from the Job Satisfaction Scale to a 3-point scale (1 = very dissatisfied and dissatisfied, 2 = neutral, 3 = satisfied and very satisfied) because some of the categories in the 5-point scale had low counts and did not meet the requirements of a chi-square test.

**Table 1:** Comparison of overall job satisfaction according to respondent characteristics

Variable	Overall job satisfaction		*p*-value
	Mean	SD	
Determinants of job satisfaction			
*PCPs’ characteristics*			
Age (years)^a^			0.005^*1^
≤ 30	2.98	0.39	
> 30 to ≤45	3.14	0.59	
> 45 to ≤60	3.30	0.59	
> 60	3.41	0.66	
Gender			0.278^2^
Male	3.25	0.62	
Female	3.16	0.51	
Length of work in main practice (years)^b^			0.004^*3^
≤ 1	3.07	0.57	
> 1 to ≤5	3.27	0.55	
> 5 to ≤15	3.10	0.51	
> 15	3.49	0.44	
*Job and practice characteristic*			
Type of practice^c^			< 0.001^*1^
Health centre	2.92	0.55	
Health centre with inpatient care	2.90	0.43	
PHC clinic	3.22	0.49	
Solo practice	3.46	0.55	
Average total income per month for the last 3 months^d^			< 0.001^*3^
≤ Rp. 3.000.000	3.27	0.50	
> Rp 3.000.000 to ≤Rp 10.000.000	3.06	0.53	
> Rp 10.000.000 to ≤Rp 20.000.000	3.22	0.53	
> Rp 20.000.000 to ≤Rp 30.000.000	3.35	0.54	
> Rp 30.000.000 to ≤Rp 40.000.000	3.44	0.40	
> Rp 40.000.000 to ≤Rp 50.000.000	3.65	0.38	
> Rp 50.000.000	3.83	0.68	
Average number of JKN patients examined per day			0.535^1^
0 to ≤40	3.18	0.54	
> 40 to ≤80	3.23	0.54	
> 80	3.05	0.73	
Average number of privately insured and FFS patients examined per day			0.040^*1^
0 to ≤40	3.20	0.53	
> 40 to ≤80	2.92	0.62	
> 80	3.49	0.74	
Performing management task			< 0.001^*4^
Yes	3.33	0.56	
No	3.05	0.51	
Perception of and experience with patients			
Perception of and experience with patients			< 0.001^*4^
Bad	2.86	0.44	
Good	3.37	0.51	

^1^ANOVA

^2^Mann-Whitney U test

^3^Kruskal-Wallis H test

^4^t-test

^a^Statistical significance: p <  0.05 for > 45 to ≤60 versus < 30

^b^Statistical significance: *p* <  0.05 for > 15 versus < 1 year and > 15 versus > 5 to ≤15

^c^Statistical significance: p <  0.001 for solo practice versus health centre and solo practice versus health centre with inpatient care; *p* <  0.05 for solo practice versus PHC clinic, PHC clinic versus health centre, and PHC clinic versus health centre with inpatient care

^d^ Statistical significance: p <  0.05 for Rp 3.000.000 to ≤Rp 10.000.000 versus >Rp 50.000.000

**Table 2:** Comparison of scores for PCP job satisfaction aspects between practice types^a^

No	Aspects of job satisfaction^b^	*p* value
1	Physical working condition	< 0.001*
2	Hours of work	0.003^c^*
3	Workload/task as JKN physicians	0.128
4	Relationship with JKN patient	< 0.001*
5	Relationship with colleagues/ co-worker	0.203^c^
6	Relationship with BPJS for Health	< 0.001*
7	Role of health authority and professional association about JKN	0.013*
8	Income	0.033*
9	Freedom of working method /Autonomy	0.003*
10	Recognition for good work	< 0.001*
11	Opportunity to use abilities	0.036*
12	Adequacy of your previous education with your job	< 0.001*
13	Providing health care for patient with 144 diagnoses	< 0.001*
14	Referral in JKN	< 0.001*
15	Health service standard for JKN patient	0.004*
16	JKN programme	< 0.001*
17	Capitation system for PHC	< 0.001*
18	JKN policy as a whole	0.001*
19	Social status for being a physician	0.022*^c^

^a^Type of practices: health centre, health centre with inpatient care, PHC clinic and solo practice physician

^b^Satisfaction with aspects of job satisfaction in 3-scale (very dissatisfied and dissatisfied, neutral and very satisfied and satisfied), except c

^c^Satisfaction score in 2-scale due to the low count in some cells

* *p* < 0.05 (2-tailed), chi-squared (Mantel Haenszel linear-by-linear) test

We confirmed the normality of the distribution by using the Shapiro–Wilk test [35] and the homogeneity of variance using Levene’s test. We compared overall job satisfaction among different respondents’ characteristics and PCPs’ intention to leave their practice using the t-test and an analysis of variance (ANOVA) for normally distributed variables or the Mann-Whitney U test and Kruskal-Wallis H test, both with Bonferroni-corrected multiple comparisons for the post hoc test, when the normality assumption was violated. In this analysis, we treated all respondents’ characteristics as categorical variables.

For ordinal scaled variables, the Cochran–Mantel–Haenszel linear-by-linear chi-squared test was applied [36]. This test was used to compare differences in PCPs’ scores for the different aspects of job satisfaction according to practice type.

To identify the predictors of PCP satisfaction, we used univariate and multiple linear regression analyses. We treated the variables of age, length of work in main practice, average number of JKN patients examined per day, and average number of private insurance and FFS patients examined per day as continuous. We chose a liberal *p*-value (*p* <  0.3) for the univariate analysis to avoid excluding any variables that might indicate a significant association in the multiple linear regression analysis [37]. Based on this liberal *p*-value, the variables identified by the univariate linear regression for inclusion in the multiple regression model were age, gender, length of work in main practice, type of practice, average total income per month, average number of privately insured and FFS patients examined, performing management tasks, and perception of and experience with patients. We excluded the average number of JKN patients examined per day because the *p*-value of this variable was more than the liberal p-value. Subsequently, we performed the multivariate regression including these variables, using stepwise variable selection by p-value. The stepwise procedure was conducted manually by excluding variables found to not be significant in each step of the analysis (backward selection). We retained the liberal p-value in deciding the inclusion of variables in step 2 of the regression analysis. Based on the results of step 1, we excluded the two variables: “length of work in main practice” and “average number of private insurance and FFS patients examined.” In step 3 of the multiple regression analysis, we began using a stricter p-value, *p* <  0.05. The final predictors of job satisfaction in step 3 were type of practice, performance of management task, and perception of and experience with patients.

To estimate the PCPs’ intention to leave their practice, we applied logistic regression analysis for the closed-ended questions with binary outcome on that sub-section of the questionnaire. All the statistical analyses were conducted with IBM SPSS Statistics 24. We also conducted inductive coding on PCPs’ answers to the open-ended questions in the sub-section evaluating the PCPs’ intention to leave their practice—that is, their reasons for intending to leave their place of practice—using NVivo 11.

## Results

### Personal characteristic and overall job satisfaction

Most respondents were female (70.3%), aged more than 30 until 45 years (47.1%) and had more than one to five years of experience in their current practice (39.9%). The majority of respondents worked as physicians in a PHC clinic (46.7%), received a monthly income of about more than Rp. 3,000,000–10,000,000 (US$210–US$700) (51.4%), and treated up to 40 JKN (69.9%) and non-JKN patients (89.9%) per day. About 51.4% of the respondents did not have managerial tasks, and 63.8% had positive perceptions of and experiences with patients.

Table 1 provides a comparison of overall job satisfaction according to respondents’ characteristics.

PCPs aged more than 45–60 years and who had more than 15 years of experience in their main practice were more likely to be satisfied than were PCPs up to 30 years of age and who had up to one or more than 5–15 years of experience (*p* <  0.05). We also found that PCPs with a monthly income of more than Rp. 50,000,000 (US$3500) and who worked in a PHC clinic or a solo practice had higher satisfaction levels than did PCPs who earned more than Rp. 3,000,000–10,000,000 (US$210–700) per month and who worked in other types of practices (p <  0.05). PCPs who had managerial tasks and reported positive perceptions of and experiences with patients also had higher satisfaction than did PCPs who did not have those conditions (*p* <  0.05).

### PCP satisfaction levels according to specific aspects

Figure 1 shows the satisfaction levels for the 19 different aspects of job satisfaction. The three aspects that most PCPs were very dissatisfied or dissatisfied with were the new referral rules due to the reform (54.0%), the introduction of new health service standards for JKN patients (51.1%), and implementation of JKN policy (39.1%). By contrast, PCPs were satisfied or extremely satisfied with their relationships with colleagues/co-workers (79.3%), working hours (70.3%), and physical working conditions (66.7%). The overall PCP satisfaction score was 3.19 out of 5.00.

Table 2 shows the differences in PCPs’ scores for the different job satisfaction aspects according to the type of practice (health centre, health centre with inpatient care, PHC clinic, and solo practice). The result showed these groups differed in their scores for almost all job satisfaction aspects, except for those for workload and relationship with colleagues.

### Predictors of PCP satisfaction

Table 3 shows the main predictors of overall job satisfaction based on a simple and multivariable linear regression analysis. In the univariate analysis, only the average number of JKN patients examined per day was not significantly associated with overall job satisfaction; thus, we included all variables except for that one in the first step of stepwise multivariate analysis. At the third step of the multivariate analysis, we found that working in a solo practice was associated with higher satisfaction than was working at a health centre (*p* = 0.001) or a health centre with inpatient care (*p* = 0.004). Moreover, physicians with managerial tasks and positive perceptions of and experiences with patients were more satisfied than were those without managerial tasks (*p* <  0.001) and who had poor perceptions of and experiences with patients (*p* <  0.001).

**Table 3:** Predictor of overall PCP satisfaction

Variable	Simple linear regression (unadjusted)		Multiple linear regression		
	coef	Sig	coef	SE	Sig
*PCPs’ characteristics*					
Age (per 5-year increase)	0.044	0.005^#^			
Gender (ref: Male)		0.260^#^			
Female	−0.081				
3 Length of work in main practice (per 5-year increase)	0.042	0.122^#^			
*Job and practice characteristic*					
Type of practice (ref: Solo practice)		0.000^#^			
Health centre	−0.544		− 0.310	0.092	0.001*
Health centre with inpatient care	−0.564		−0.313	0.108	0.004*
Clinic	−0.244		−0.780	0.076	0.311
Average total income per month for last 3 months^d^ (ref: ≤Rp. 3.000.000 (ref)		0.000^#^			
> Rp 3.000.000 to ≤Rp 10.000.000	−0.212				
> Rp 10.000.000 to ≤Rp 20.000.000	−0.049				
> Rp 20.000.000 to ≤Rp 30.000.000	0.081				
> Rp 30.000.000 to ≤Rp 40.000.000	0.165				
> Rp 40.000.000 to ≤Rp 50.000.000	0.383				
> Rp 50.000.000	0.558				
Average number of JKN patients examined per day	−0.001	0.322			
Average number of private insurance and FFS patients examined per day	−0.003	0.056^#^			
Performing management tasks (ref: Yes)		0.000^#^			
No	−0.282		−0.235	0.061	0.000*
*Perception of and experience with patients*					
Perception of and experience with patients (ref: Bad)		0.000^#^			
Good	0.514		0.467	0.058	0.000*

# *p* < 0.3

*p < 0.05

### Intention to leave practice

Table 4 shows a comparison of overall job satisfaction according to the intention to leave their practice between the groups. Respondents who were willing to continue working as PCPs for JKN patients (89.1%) had a significantly higher level of satisfaction than did PCPs who intended to leave their practices (*p* = 0.005). Furthermore, the logistic regression analysis showed that higher job satisfaction might prevent PCPs from leaving JKN practices. For every point increase in job satisfaction, the odds of the intention to leave the practice decreased by 67.7% (OR = 0.323, *p* = 0.004).

**Table 4:** Intention to leave practice

Variable	Intention to leave practice as PCPs for JKN	
	No	Yes
Job satisfaction		
Mean	3.22	2.91
SD	0.55	0.4
p		0.005*
Logistic regression		
OR		0.323
95% CI		0.149–0.700
*p*-value		0.004^#^

*0.05 (2-tailed), Kruskal-Wallis test

^#^0.05 (2-tailed), Logistic regression

The analysis of the open questions revealed that the reasons PCPs felt obliged to stay at their practices were that they perceived themselves as civil servants and believed that treating JKN patients was their duty.

## Discussion

Most PCPs were dissatisfied with the new referral rules within the system. This result differs from a study in Iran showing that PCPs were generally satisfied with the referral system [17]. In Indonesia, before the reform, the tiered referral system – running from primary to secondary and tertiary care – was not optimally implemented [4, 38]. According to a BPJS for Health report, the referral rate was 15.29% in the 1st quarter of 2015 [39]. The reasons for the poor referral was attributed to problems with financing, physician competency, and a lack of medical devices [12]. Following the reform, the implementation of tiered referral system became more rigorous. PCPs must be able to diagnose and manage 144 diseases completely, as stated in the ‘Competency standards of Indonesian physician guidelines’ [40]; however, this is not possible in practice. PCPs can refer patients with one of the 144 listed diagnoses to higher level healthcare facilities if the patient meets the Time-Age-Complication-Comorbidity (TACC) minimum criteria, of if the available health facilities are inadequate [12, 41]. However, in a separate qualitative study we conducted (not as part of this paper), we found that if PCPs referred patients with any of the 144 listed diagnoses, they received poor marks in evaluations by BPJS for Health.

The challenges of the implementation of the JKN health service standards, such as the competency standards, JKN formulary, and diagnosis examination coverage, resulted in PCPs’ dissatisfaction. These findings are possibly linked to the lower number of medicines in the new national formulary when compared to the formulary under PT. Askes (the previous public health insurance for civil servants), and the limitations of diagnosis examination coverage. A similar phenomenon occurred in the United States (US), wherein physicians became dissatisfied with the limitations of health treatment coverage put in place by payers. Numerous high-income countries have not yet achieved 100% on all three dimensions of universal coverage—population, cost, and services [42]. Surprisingly, Thailand, despite being a lower-income country, has seen a rapid increase in health service coverage index—it is now 75, making it much higher than Indonesia’s 49 [42, 43].

PCPs were also dissatisfied with the JKN policy. This finding is similar to previously noted satisfaction rates in South Korea, where about 71.5% of physicians in all healthcare facilities were unsatisfied with the national health insurance (NHI) policy [44], which was established in 1963 and extended to cover the entire population in 1989 [45]. In Germany—wherein most of the population is covered by public health insurance [46]—about 82% of PCPs considered the health system to be in need of a major change. This perception might relate to the reimbursement system reform implemented in 2009 [47]. However, another study in the US revealed that most physicians in Wisconsin, where private health insurance covers most citizens, were not satisfied with the health system [48, 49] and encouraged the government to establish the NHI [49].

The three aspects with which PCPs showed the most dissatisfaction were all related to the JKN reform. This needs consideration, particularly because this study comes only two years after JKN implementation and a new BPJS for Health regulation on performance-based capitation in the study area [11].

One of the satisfying aspects was PCPs’ relationships with their colleagues; namely physicians, dentists, nurses, midwives, pharmacists, laboratory analysts, and administrative staff. Other studies have also shown that physicians in many countries tend to be satisfied with their relationships with both colleagues and fellow workers [32, 50, 51].

Most of the PCPs reported being satisfied with their working hours and physical working conditions. This finding is the same as that of an earlier study in Malaysia, in which physicians reported being satisfied with these two aspects [50]. A possible reason that PCPs reported being satisfied with their working hours is that we only asked about the working hours in their main place of practice, rather than the overall working hours (which would be greater for those working in two or three places). As to their satisfaction with physical working conditions (i.e. practice location, working room/building, and medical and non-medical equipment), we might attribute this to the fact that the survey was conducted in Java Island, which has better infrastructure than does other islands.

In this study**,** PCPs working in a solo practice, who had managerial tasks, and who had a good perception and relationships with patients, tended to have higher overall job satisfaction scores. Although this finding differs from that of a previous study [52], it is consistent with the findings of another study [53] wherein solo practice physicians reported having the greatest job satisfaction level, which they attributed to their autonomy [54], the increased opportunity to employ their ability, and the possibility of higher income. In 2008, Indonesian solo practice PCP’s income were between Rp. 336,000 and Rp. 20,580,000 (US$22.6–1382.13), with an average of Rp. 5,222,346 (US$350.7) gained from capitation and FFS [19]. By contrast, the PCPs in this study had a higher income than that reported in 2008.

The physicians working in PHC clinics and health centres had lower overall satisfaction than did solo practice physicians. This might be linked to lower work control [55], especially in government-owned health centres; comparatively, those who own their own practices have higher work control and thus higher satisfaction [56]. Furthermore, physicians with managerial tasks—most solo practice physicians—had a higher levels of job satisfaction. Previous findings have shown similar results that physicians who serve as clinic directors also have higher satisfaction scores than do non-directors [57].

Health centres also tend to have a much greater number of patients compared to PHC clinics and solo practices. However, sometimes, in health centres, nurses and midwives fulfil some of PCPs’ function [58]. Furthermore, generally, there are more co-workers in health centres than in PHC clinics and solo practices, meaning that the workloads can be shared. Therefore, we cannot likely attribute the satisfaction differences between the practice types to workload differences.

PCPs with good perceptions of and experiences with patients (e.g., whether patients made unrealistic requests) to be more satisfied than did those without such perceptions or experiences. This result accords with that of an earlier study stating that physicians who believed that their patients had realistic requests had a higher level of satisfaction [30]. Furthermore, another study showed that respect from the patient was a predictor of physicians’ overall job satisfaction [59].

Job satisfaction contributes to PCPs’ intention to remain in their main place of practice as a PCP for JKN. A similar result was obtained in previous studies [15, 60]. Even though PCPs’ overall satisfaction score was moderate, most chose to keep practising in PHC facilities. This is perhaps because the majority of PCPs who worked in health centres and health centres with inpatient care were civil servants and they had to support government policy. Moreover, PCPs who worked in private practices (the majority of PHC clinics and solo practices) thought that Indonesian citizens would be JKN participants, meaning that they should follow the market trend.

### Limitations of the study

The study was conducted in a small area – just two cities on Java Island. Because Indonesia has numerous islands and cultures, and high variability in geographical conditions, we should conduct similar research in more hard-to-reach areas, such as small islands or remote regions throughout Indonesia. Furthermore, our findings only reflect respondents’ satisfaction in one practice place, even though of them worked in two or three healthcare facilities. Moreover, despite our use of various methods of data collection (to increase the response rate and reduce number of missing responses), there is the possibility of bias in our data. There is also possible participation bias, because we excluded PCPs who worked less than three months and PCPs who worked in PHC facilities with a contract with BPJS Kesehatan for less than three months. Therefore, the generalisability of this study to Indonesian PCPs’ satisfaction was limited.

## Conclusion

This study found that PCP had moderate levels of job satisfaction in two selected cities in the Java region of Indonesia. Working in solo practices, having managerial tasks, and having good perceptions of and experiences with patients contributed to higher satisfaction scores. Higher satisfaction scores could also prevent PCPs’ intention to leave their practice. To ensure that PCPs stay working in their place of practice and are satisfied with their job, PCPs require more autonomy and an opportunity to use their abilities. Moreover, the government must improve the JKN’s referral system, health service standards, and health coverage and procedure to increase Indonesian PCPs’ satisfaction.

## Additional files

Additional file 1: Questionnaire: Determining the main place of practice. (PDF 36 kb)

Additional file 2: Questionnaire: Respondents’ characteristics (PCPs’ personal characteristics, job and practice characteristics, and the PCPs’ perception and experiences with their patients. (PDF 75 kb)

Additional file 3: Questionnaire: PCP satisfaction. (PDF 28 kb)

Additional file 4: Questionnaire: Intention to leave practice. (PDF 19 kb)

## Abbreviations

ANOVA: Analysis of variance
BPJS: Badan Penyelenggara Jaminan Sosial
DHF: Dengue haemorrhagic fever
FFS: Fee-for-service
IR: Incidence rate
JKN: Jaminan Kesehatan Nasional
NHI: National Health Insurance
PCP: Primary care physician
PHC: Primary healthcare
TACC: Time-age-complication-comorbidity
