# Supplementary material for: Primary care physicians’ satisfaction after health care reform: a cross-sectional study from two cities in Central Java, Indonesia
Source: BMC Health Serv Res. 2019 May 8;19:290. doi: 10.1186/s12913-019-4121-2 (PMC6505224; doi:10.1186/s12913-019-4121-2)
Supplement: Supplementary file 1 — Questionnaire: Determining the main place of practice. (PDF 36 kb) [file 12913_2019_4121_MOESM1_ESM.pdf]

Additional file 1, Questionnaire: Determining the main place of practice.

Determining the main place of practice

(only for physician who works in more than one PHC facilities which have MoU with Semarang Main Branch Office of BPJS for Health)

Please fill the blank or choose the options by thick (√) based on your condition in the following questions.

Type of practice (only PHC facilities which have MoU with Semarang Main Branch Office of BPJS for Health)		Public health centre/PHC Clinic/Solo practice*		Public health centre/PHC Clinic/Solo practice*		Public health centre/PHC Clinic/Solo practice*	
Name and Addres of Practice		1. ____________________		2. ____________________		3. ____________________	
No	Questions		Scr		Scr		Scr
1.	Working period as physician in the place of practice (included before JKN and ASKES era)	☐ a. 0 up to ≤ 3 months	*0*	☐ a. 0 up to ≤ 3 months	*0*	☐ a. 0 up to ≤ 3 months	*0*
		☐ b. 3 months up to ≤ 6 months	*1*	☐ b. 3 months up to ≤ 6 months	*1*	☐ b. 3 months up to ≤ 6 months	*1*
		☐ c. > 6 months up to ≤ 2 years	*2*	☐ c. > 6 months up to ≤ 2 years	*2*	☐ c. > 6 months up to ≤ 2 years	*2*
		☐ d. > 2 years	*3*	☐ d. > 2 years	*3*	☐ d. > 2 years	*3*
2.	Total working hours in a week (include on call)	☐ a. ≤ 20 hours	*1*	☐ a. ≤ 20 hours	*1*	☐ a. ≤ 20 hours	*1*
		☐ b. > 20 hours up to ≤ 40 hours	*2*	☐ b. > 20 hours up to ≤ 40 hours	*2*	☐ b. > 20 hours up to ≤ 40 hours	*2*
		☐ c. > 40 hours up to ≤ 50 hours	*3*	☐ c. > 40 hours up to ≤ 50 hours	*3*	☐ c. > 40 hours up to ≤ 50 hours	*3*
		☐ d. > 50 hours	*4*	☐ d. > 50 hours	*4*	☐ d. > 50 hours	*4*
3.	Do you perform managerial task except working as a physician practice?	☐ a. No	*1*	☐ a. No	*1*	☐ a. No	*1*
		☐ b. Yes, having some managerial task (include JKN)	*2*	☐ b. Yes, having some managerial task (include JKN)	*2*	☐ b. Yes, having some managerial task (include JKN)	*2*
		☐ c. Yes, as a manager	*3*	☐ c. Yes, as a manager	*3*	☐ c. Yes, as a manager	*3*
		☐ d. Yes, as an owner and not a manager	*3*	☐ d. Yes, as an owner and not a manager	*3*	☐ d. Yes, as an owner and not a manager	*3*
		☐ e. Yes, as a manager and an owner	*4*	☐ e. Yes, as a manager and an owner	*4*	☐ e. Yes, as a manager and an owner	*4*
**Score**							

*) *Cross the options that does not fit*

Result (filled by enumerator)

Main place of practice : ______________________________
