# Supplementary material for: Primary care physicians’ satisfaction after health care reform: a cross-sectional study from two cities in Central Java, Indonesia
Source: BMC Health Serv Res. 2019 May 8;19:290. doi: 10.1186/s12913-019-4121-2 (PMC6505224; doi:10.1186/s12913-019-4121-2)
Supplement: Supplementary file 2 — Questionnaire: Respondents’ characteristics (PCPs’ personal characteristics, job and practice characteristics, and the PCPs’ perception and experiences with their patients. (PDF 75 kb) [file 12913_2019_4121_MOESM2_ESM.pdf]

Additional file 2, Questionnaire: Respondents' characteristics (PCPs' personal characteristics, job and practice characteristics, and the PCPs' perception and experiences with their patients.

Please fill the questionnaire based on working condition in your PHC facilities which have MoU with Semarang Main Branch Office of BPJS for Health (Semarang Municapility and Demak Regency)

A. *If you work at 1 PHC facility which have MoU with Semarang Main Branch Office of BPJS for Health, please fill the questionnaire based on your working condition in the PHC facility.*
B. *If you work at 2-3 PHC facilities which have MoU with Semarang Main Branch Office of BPJS for Health, please fill the questionnaire based on the result of "Determining the main place of practice" questionnaire.*

Name of Phsyician :____

Main place of practice :____

(Based on the result of "Determining the main place of practice ")

The PHC facilities had started MoU with BPJS for Health from (month/year) : ____________/_______

Name of Enumerator :____

A. PCPs' personal characteristics

Please fill the blank or choose the options by thick (√) based on your condition in the following questions

1. Name (with education title) :_ ____
2. Date of Birth (DD/MM/YYYY) : ____/____/ ________
3. Gender : ☐ 1. Male

   ☐ 2. Female
4. Length of work as physician in main place of practice : ________ years ________ months

B. Job and practice characteristics

Please fill the blank or choose the options by thick (√) based on your condition in ***your main place practice*** *in the*

5. Type of practice place *(circle the suitable one)* : 1. Public health centre (Non in-patient care/In-patient care)*
   2. PHC Clinic
   3. Solo practice
6. Average of total income for last 3 months : ☐ 1. ≤ Rp. 3.000.000,-

   ☐ *2. > Rp. 3.000.000,- up ≤ Rp. 10.000.000,-*

   ☐ *3. > Rp. 10.000.000,- up ≤ Rp. 20.000.000,-*

   ☐ *4. > Rp. 20.000.000,- up ≤ Rp. 30.000.000,-*

   ☐ *5. > Rp. 30.000.000,- up ≤ Rp. 40.000.000,-*

   ☐ *6. > Rp. 40.000.000,- up ≤ Rp. 50.000.000,-*

   ☐ *7. > Rp. 50.000.000,-*
7. Average number of JKN patients examined per day : __________ patients
8. Average number of private insured and Fee for Service (FFS) patients (non-JKN) examined per day : __________ patients
9. Performing managerial task (include JKN) ☐ 1. Yes

   ☐ 2. No

* *circle the suitable one*

C. PCPs' perception and experiences with their patients

Please choose and thick (√) the options based on your condition in ***your main place of practice*** *in the following questions.*

a. Do you feel the JKN patients and/or patients' family appreciate you?

☐ 1. Yes
☐ 2. Sometimes
☐ 3. No

b. Do you feel the community appreciate you?

☐ 1. Yes
☐ 2. Sometimes
☐ 3. No

c. Do you feel patients and/or patients' family follow your advice?

☐ 1. Yes
☐ 2. Sometimes
☐ 3. No

d. Do you feel JKN patients and/or patients' family have unrealistic expectations?

☐ 1. Yes
☐ 2. Sometimes
☐ 3. No

e. Do you feel JKN patients and/or patients' family has difficulty to understand about JKN procedure for getting health services?

☐ 1. Yes
☐ 2. Sometimes
☐ 3. No

f. Have your JKN patients and/or patients' family ever complained against the service that you provide?

☐ 1. Yes
☐ 2. No

g. Have your JKN patients and/or patients' family ever been angry to you?

☐ 1. Yes
☐ 2. No

h. Have your JKN patients and/or patients' family ever been angry with you?

☐ 1. Yes
☐ 2. No

i. Did you ever get the physical violence of your patients?

☐ 1. Yes
☐ 2. No
