# Supplementary material for: Primary care physicians’ satisfaction after health care reform: a cross-sectional study from two cities in Central Java, Indonesia
Source: BMC Health Serv Res. 2019 May 8;19:290. doi: 10.1186/s12913-019-4121-2 (PMC6505224; doi:10.1186/s12913-019-4121-2)
Supplement: Supplementary file 3 — Questionnaire: PCP satisfaction. (PDF 28 kb) [file 12913_2019_4121_MOESM3_ESM.pdf]

Additional file 3: Questionnaire: PCP satisfaction.

D. Work Satisfaction

Please indicate your level of satisfaction on each statement of aspects/facets of job satisfaction by thick (√) the colum based on your condition in ***your main place of practice*** *.*

What is level your satisfaction in …

No.	Aspects	Extremely not satisfied	Not satisfied	Neutral	Satisfied	Extremely satisfied
		(1)	(2)	(3)	(4)	(5)
1	Physical working condition					
2	Hours of work					
3	Workload/task as JKN physicians					
4	Relationship with JKN patient					
5	Relationship with colleagues/ co-worker (physician, dentist/networking dentist, nurse, midwives/networking midwives, pharmacist, laboratory analist, administrative staffs, etc.)					
6	Relationship with BPJS for Health					
7	Role of health authority and professional association about JKN					
8	Income					
9	Freedom of working method /Autonomy					
10	Recognition for good work					
11	Opportunity to use abilities					
12	Adequacy of your previous education with your job					
13	Providing health care for patient with 144 diagnoses					
14	Referral in JKN					
15	Health service standard for JKN patient, for instance 144 diagnoses, formularium and coverage of diagnosis examination					
16	JKN programme, for instance home visit, medical history screening, Chronic diseases management programme (PROLANIS),etc.					
17	Capitation system for PHC					
18	JKN policy as a whole					
19	Social status for being a physician					
