# Supplementary material for: Primary care physicians’ satisfaction after health care reform: a cross-sectional study from two cities in Central Java, Indonesia
Source: BMC Health Serv Res. 2019 May 8;19:290. doi: 10.1186/s12913-019-4121-2 (PMC6505224; doi:10.1186/s12913-019-4121-2)
Supplement: Supplementary file 4 — Questionnaire: Intention to leave practice. (PDF 19 kb) [file 12913_2019_4121_MOESM4_ESM.pdf]

Additional file 4: Intention to leave practice.

E. Intention to leave practice

Please give check sign (√) on the options that appropriate with you carried out and explain the reason.

I. Intention to leave practice as PHC physician for JKN

1. Do you want to leave your practice as JKN PHC physician in the main place practice?

☐ a. Yes

Reason

____

____

☐ b. No

Reason

____

____
